# Supplementary material for: Drug reinforcement impairs cognitive flexibility by inhibiting striatal cholinergic neurons
Source: Nat Commun. 2023 Jun 30;14:3886. doi: 10.1038/s41467-023-39623-x (PMC10313783; doi:10.1038/s41467-023-39623-x)
Supplement: Supplementary file 1 — Supplementary Information [file 41467_2023_39623_MOESM1_ESM.pdf]

Drug Reinforcement Impairs Cognitive Flexibility by Inhibiting Striatal Cholinergic Neurons

Supplementary Information

Supplementary Figures

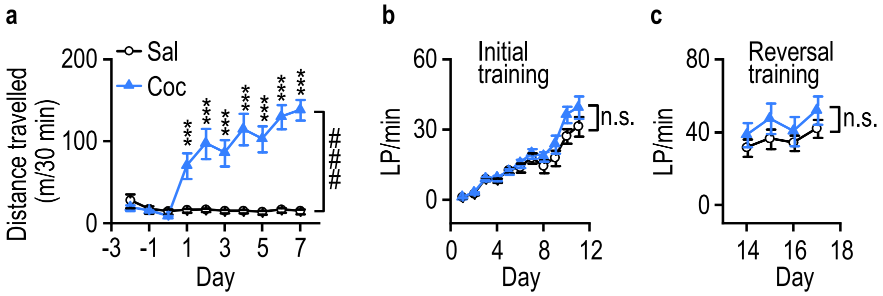

Supplementary Fig. 1. Cocaine-induced hyperlocomotion but no changes in lever press rate during initial and reversal training in mice.

a, Repeated cocaine (Coc, blue; 15 mg/kg) but not saline (Sal, black) injections caused hyperlocomotion in C57/BL6 wild-type mice; $^{***}p < 0.001$, $^{\#\#\#}p < 0.001$. **b, c,** There was no difference in lever press (LP) rate between the saline and cocaine groups during initial (b) and reversal training (c). n.s. (not significant; b, c). Two-way RM ANOVA followed by Tukey *post-hoc* test (a, b, c). n = 11 (a-c, Sal), 13 (a-c, Coc). Data are presented as mean values +/- SEM. Source data are provided as a Source Data file.

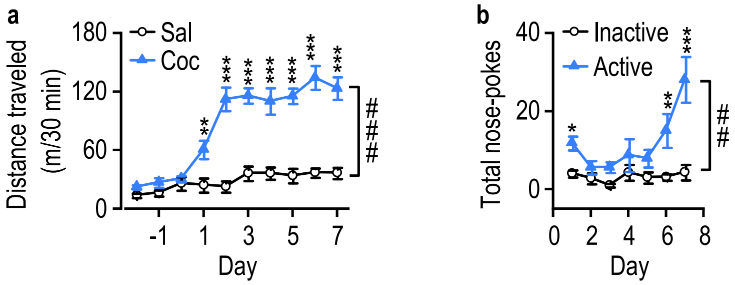

Supplementary Fig. 2. Cocaine-induced hyperlocomotion and cocaine IVSA in ChAT-eGFP mice.

a, Repeated cocaine (Coc, blue) but not saline (Sal, black) injections caused hyperlocomotion in ChAT-eGFP mice; $^{**}p < 0.01$, $^{***}p < 0.001$, $^{\#\#\#}p < 0.001$. **b,** Total active versus inactive nose-pokes to receive intravenous cocaine infusions across training sessions in ChAT-eGFP mice; $^{*}p < 0.05$, $^{**}p < 0.01$, $^{***}p < 0.001$, $^{\#\#}p = 0.001$. Two-way RM ANOVA followed by Tukey *post-hoc* test (a, b). n = 5 mice (a, Sal), 5 (a, Coc), 8 (b). Data are presented as mean values +/- SEM. Source data are provided as a Source Data file.

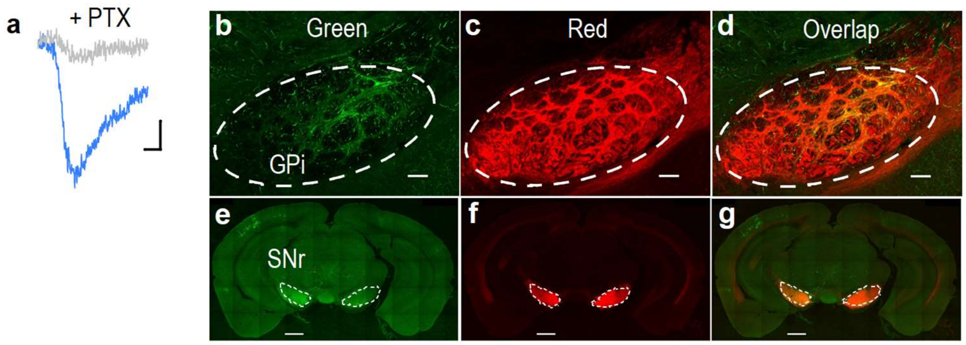

Supplementary Fig. 3. CIN-innervating dMSNs project to the GPi and SNr.

a, Sample trace of light (470 nm)-evoked PSC in a DMS CIN (blue), blocked by picrotoxin (PTX, 100 μM; grey). **b-d,** Confocal images of internal globus pallidus (GPi), channels: green (b), red (c) and overlap (d). **e-g,** Confocal images of substantia nigra pars reticulata (SNr), channels: green (e), red (f) and overlap (g). b-g share the same mice (ChAT-Cre;D1tdT mice infused with rabies-GFP) as described in Figure 3a-i. Scale bars: 10 ms, 10 pA (a); 100 μm (b-d); 1 mm (e-g). Source data are provided as a Source Data file.

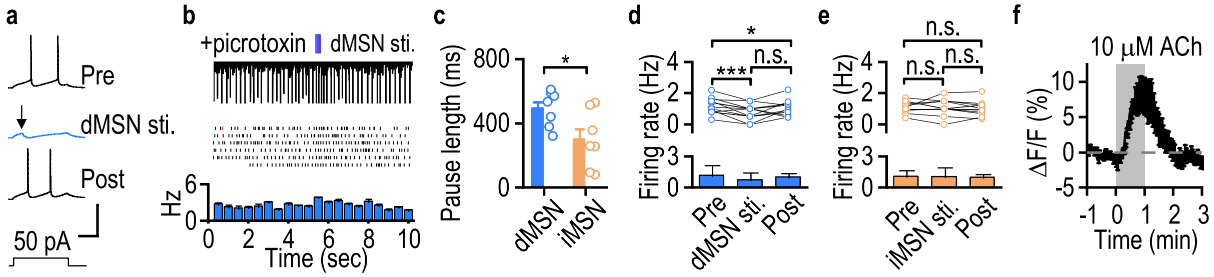

Supplementary Fig. 4. Impact of optogenetic dMSN or iMSN stimulation on CIN firing.

a, Evoked firing of a cholinergic interneuron (CIN) before (Pre, black), during (dMSN sti., blue), and after (Post, black) optogenetic stimulation (sti.) of direct pathway medium spiny neurons (dMSNs) in D1-Cre;Ai32 mice. **b,** Picrotoxin (100 μM) abolished dMSN stimulation (20 Hz, 5 ms, 5 p)-induced pause-rebound firing activity in a CIN. Top, sample firing; middle, multiple sweeps; bottom, the corresponding histogram. **c,** Summarized data demonstrating a longer pause in CIN firing after dMSN (blue) versus indirect pathway MSN (iMSN, orange) burst-stimulation in Ai32 transgenic mice; *p = 0.033. **d,** Summarized data demonstrating inhibition of CIN activity on dMSN burst stimulation (20 Hz, 5 ms, 5 p); D1-Cre;ChATeGFP mice infused with AAV-FLEX-Chrimson-tdT in the DMS; *p = 0.0236, ***p < 0.001. **e,** Summarized data demonstrating no inhibition of CIN activity on iMSN burst stimulation (20 Hz, 5 ms, 5 p); A2A-Cre;ChATeGFP mice infused with AAV-FLEX-Chrimson-tdT in the DMS. **f,** A sample live-tissue confocal recording in the DMS of a wild-type rat infused with AAV-iAChSnFR in response to bath application of acetylcholine (ACh, 10 μM). n.s. (not significant; d, e). Scale bar: 100 ms, 50 mV (a). Unpaired *t* test (c), paired *t* test (d, e). n = 7/4 (c, dMSN), 7/4 (c, iMSN), 13/3 (d), 12/3 (e). Data are presented as mean values +/- SEM. Source data are provided as a Source Data file.

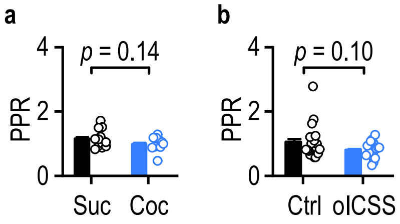

Supplementary Fig. 5. Cocaine IVSA and dMSN oICSS alter paired-pulse ratios of dMSN→CIN oIPSCs.

a, Summarized data demonstrating a trend in the reduction of paired-pulse ratios (PPR) of dMSN→CIN oIPSCs in the cocaine (Coc, blue) group as compared to the sucrose (Suc, black) group. **b,** Summarized data demonstrating a trend in the reduction of paired-pulse ratios of dMSN→CIN oIPSCs in the dMSN self-stimulation (oICSS, blue) group as compared to controls (black). Unpaired *t* test (a, b). n = 22/5 (a, Suc), 21/5 (a, Coc), 11/4 (b, Ctrl), 12/4 (b, oICSS). Data are presented as mean values +/- SEM. Source data are provided as a Source Data file.

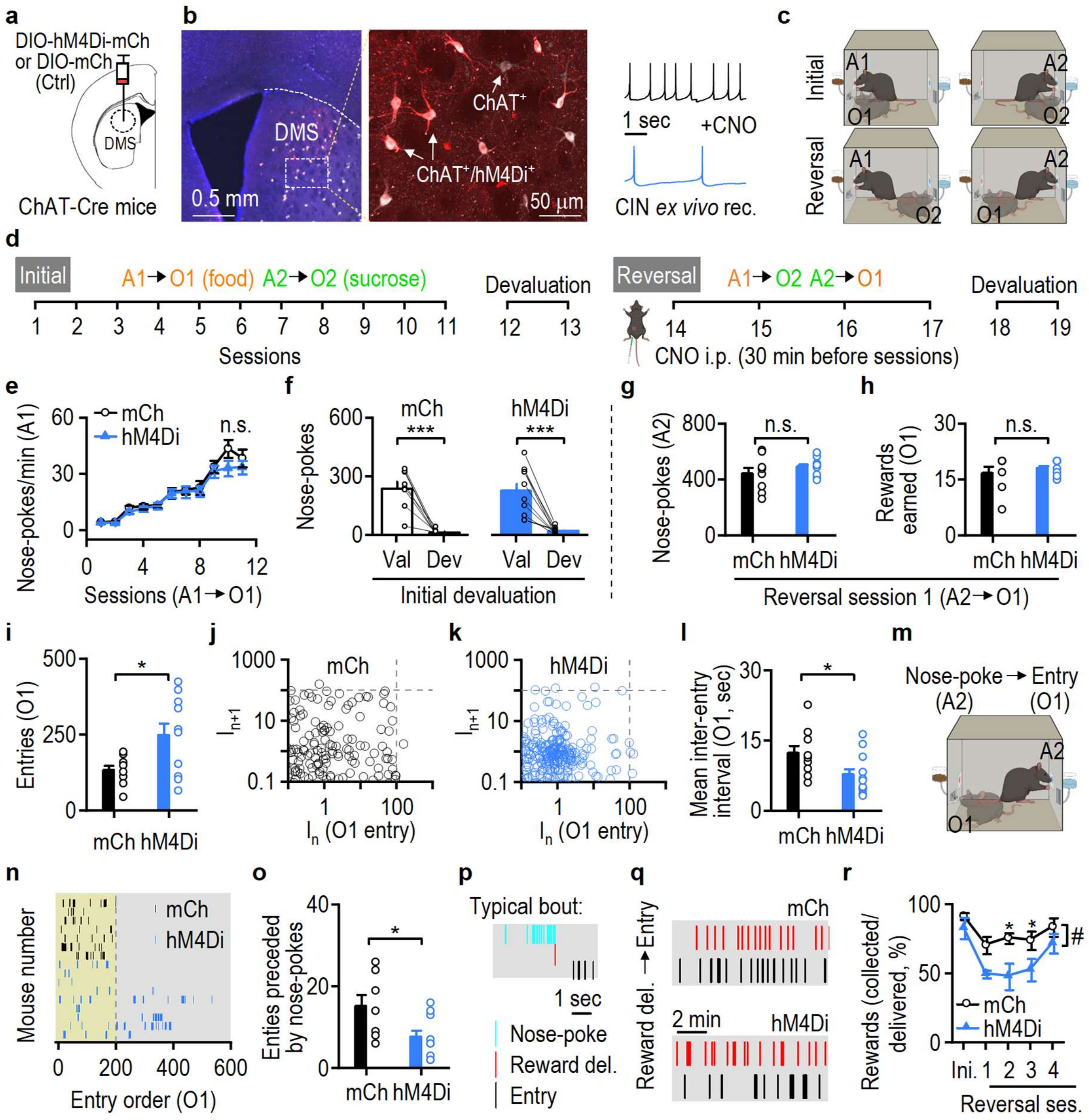

Supplementary Fig. 6. Chemogenetic inhibition of DMS CINs during reversal training impairs action-outcome association.

a, AAV-DIO-hM4Di-mCherry (blue) or AAV-DIO-mCherry (Ctrl: control, black) was infused into the dorsomedial striatum (DMS) of ChAT-Cre mice. **b,** Micrographs demonstrating hM4Di-mCherry expression in DMS cholinergic interneurons (CINs) using ChAT-staining (white) (left) and clozapine N oxide (CNO)-mediated inhibition of hM4Di^{+}

CINs (right); rec.: recording. **c, d,** Schematics and timelines of initial and reversal training. **e,** Nose-poking (A1) for food did not differ between hM4Di and mCherry (mCh) groups during initial training. **f,** Both groups were sensitive to outcome devaluation after initial training; ***$p < 0.001$. **g, h,** Reversal session 1: Similar nose-pokes (A2, for food; g) and earned food rewards (O1, h) for the two groups. **i**, Reversal session 1: food magazine entries (O1) was higher in the hM4Di group; *$p = 0.033$. **j, k,** Sample return maps of inter-entry (O1)-intervals (IAI) on reversal session 1 for mCh (j) and hM4Di (k) groups. **l,** Mean inter-entry interval was lower in the hM4Di group; *$p = 0.048$. **m,** Schematic of action-outcome association during reversal training. **n, o,** Scatter plot (n) demonstrating magazine entries (O1), which were preceded by a nose-poke (A2, within 10 s) during reversal session 1. Each row represents an animal belonging to either the hM4Di or mCh group. Summarized data (o) demonstrating fewer such nose-poke (A2)-preceded entries (O1) in the hM4Di group, within the first 200 entries; *$p = 0.042$. **p,** Schematic demonstrating nose-poking (cyan), reward delivery (del.; red) and subsequent magazine entries (red) in a typical bout. **q,** Sample time-series data demonstrating reward delivery (red) and magazine entries (black, mCh; blue, hM4Di) during reversal session 1. **r,** Summarized data demonstrating reduced percentage of rewards collected over total rewards delivered in the hM4Di group (blue) across reversal training sessions (Ses.); *$p < 0.05$, $^{\#}p = 0.017$. Val: valued, Dev: devalued, Ini: Last day of initial learning. n.s. (not significant; e, g, h). Paired *t*-test (f); unpaired *t*-test (g-i, l, o, r). n = 9 (mCherry); 9 (hM4Di). Data are presented as mean values +/- SEM. Source data are provided as a Source Data file.

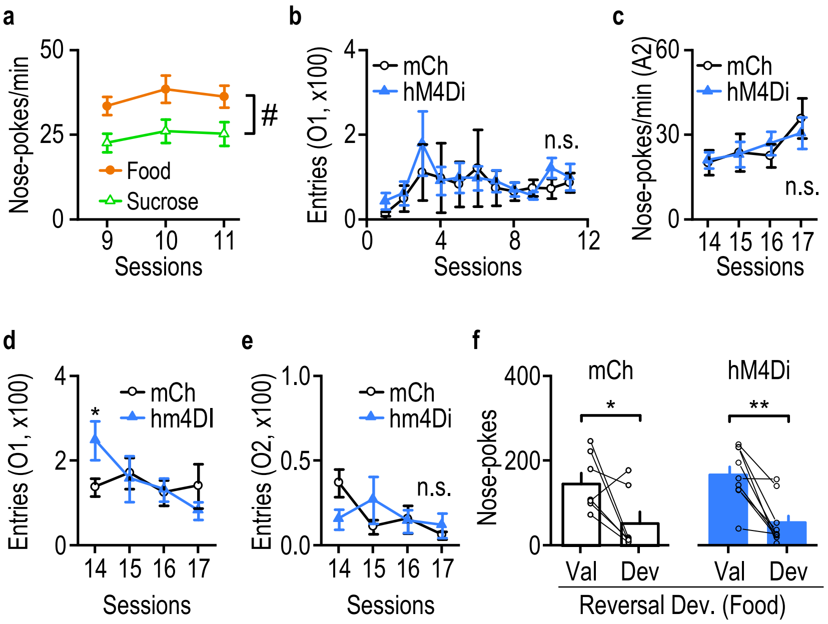

Supplementary Fig. 7. Impact of chemogenetic CIN inhibition on the initial and reversal training of food and sucrose rewards.

a, Combined data from mCherry (mCh) and hM4Di groups demonstrating that the mice preferred to nose-poke for food (orange) over sucrose (green), $^{\#}p = 0.019$. **b,** Magazine entries for food (O1) for the mCh (black) and hM4Di (blue) groups did not differ during initial training. **c,** Nose-pokes/min did not differ between groups during reversal training. **d,** Higher food magazine entries (O1) for the hM4Di group than mCherry controls on the 1st reversal training session; $^{*}p = 0.0496$. **e,** Sucrose magazine entries (O2) did not differ between groups during reversal training. **f,** Both groups were sensitive to food devaluation (Dev.) after reversal training; $^{*}p = 0.054$, $^{**}p = 0.002$. Val: valued (grey); Dev: devalued (black). n.s. (not significant; b, c, e). Two-way RM ANOVA followed by Tukey *post hoc* test (a, b, c, e), unpaired *t* test (d), paired *t* test (f). n = 9 (a-f, mCh), 9 (a-f, hM4Di). Data are presented as mean values +/- SEM. Source data are provided as a Source Data file.

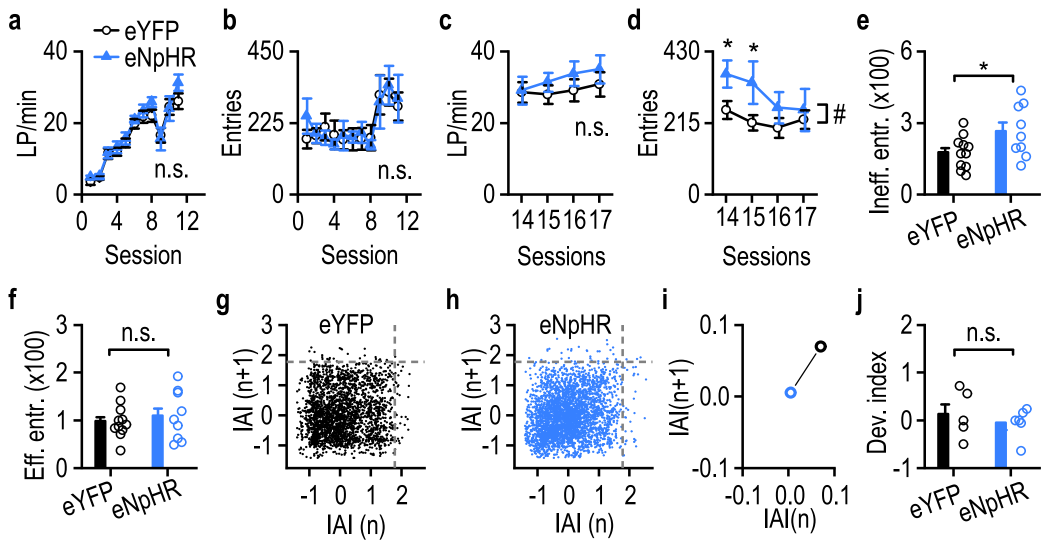

Supplementary Fig. 8. Impact of optogenetic CIN inhibition on reversal learning.

a, b, Lever-press (LP) rate and magazine entries (Entries) of eYFP (Control, black) and eNpHR (Halorhodopsin, blue) groups during initial training. **c, d,** Lever-press rate and magazine entries of eYFP and eNpHR groups during reversal training; *$p < 0.05$, $^{\#}p = 0.06$. **e, f,** Summarized data demonstrating increased ineffective (Ineff.) magazine entries (e) with no change in effective (Eff.) entries (f) for the eNpHR group as compared to eYFP controls; *$p = 0.038$. **g, h,** Return maps of inter-action intervals (IAI) between magazine entries for the control (g) and eNpHR (h) groups on the first day of reversal training (pooled group data). Each data point (x, y) represents the time difference between the previous (x) and the next time point of magazine entry (y). **i,** Plot illustrating the centers of the data points in (g, h) for the control and eNpHR groups. **j,** No difference in devaluation (Dev.) index for the 2nd devaluation was observed between the eYFP and eNpHR groups when CIN inhibition was paired to lever presses, rather than reward deliveries, during reversal training. n.s.(not significant; a, b, c, f, j). Two-way RM ANOVA (a, b, c, d), unpaired *t* test (e, f, j), n = 8 (a-i, eYFP), 8 (a-i, eNpHR), 5 (j, eYFP), 6 (j,

eNpHR). Data are presented as mean values +/- SEM. Source data are provided as a Source Data file.

Supplementary Tables

Supplementary Table 1: Virus Information

Virus	Vendor	Lot #	Titer
AAV8-FLEX-TVA-mCherry	UNC	AV5008B	5.4 e12
AAV8-FLEX-RG	UNC	AV5005F	1.8 e12
Rabies-GFP	Salk	N/A	2.04 e8
AAV-FLEX-Chrimson-tdTomato	UNC	AV5844	4.1 e12
pAAV-Ef1a-double floxed-hChR2(H134R)-EYFP-WPRE-HGHpA	Addgene	v32674	1 e13
AAV-FLEX-Chronos-GFP	UNC	AV5843	5.3 e12
AAV5-EF1a-DIO-hM4Di-mCherry	UNC	AV4622C	4 e12
AAV5-EF1a-DIO-mCherry	UNC	AV4633	6 e12
pAAV-FLEX-ArchT-tdTomato	Addgene	v24870	1.3 e13
AAV-CAG-FLEX-tdTomato	UNC	AV4912B	5.5 e12
rAAV5/EF1a-DIO-eNpHR3.0-eYFP	UNC	AV5237	4 e12
AAV-DIO-eYPF	UNC	AV4579	3 e12
AAV9- CAG-iAChSnFR	Addgene	137955	1 e13

Supplementary Table 2: Mouse Information

ID	Genotype	Vendor	Lot #
1	C57BL/6J	Jackson Laboratories	00664
2	ChAT-Cre($^{+}$/$^{+}$)	Jackson Laboratories	028861
3	deltaneoChAT-Cre($^{+}$/$^{+}$)	Jackson Laboratories	031661
4	ChATeGFP($^{+}$/$^{+}$)	Jackson Laboratories	007902
5	Ai14($^{+}$/$^{+}$)	Jackson Laboratories	007914
6	D1tdTomato($^{+}$/$^{-}$)	Jackson Laboratories	016204
7	Ai32($^{+}$/$^{+}$)	Jackson Laboratories	012569
8	Drd1-Cre($^{+}$/$^{-}$)	MMRRC	29178
9	Ai167($^{+}$/$^{-}$)	Allen Brain Institution	N/A
10	A2A-Cre($^{+}$/$^{-}$)	MMRRC	036158-UCD

Supplementary Table 3: Rat Information

ID	Genotype	Vendor	Lot #
1	D1-Cre(+/-)	RRRC	856
2	ChAT-Cre(+/-)	RRRC	658
3	tdTomato/Hom-KI(+/+)	Envigo Horizon	TGRL9660

Supplementary Discussion

The inhibitory DREADD, hM4Di, or mCherry (control) was selectively expressed in DMS CINs of ChAT-Cre mice (Supplementary Fig. 6a, b). Mice were then trained on a two-action-outcome reversal learning task (Supplementary Fig. 6c, d). During initial training, mice nose-poked on the left port (A1) for food pellets (O1; A1→O1) and nose-poked on the right port (A2) for sucrose (O2; A2→O2). Task contingencies were switched (A1→O2; A2→O1) during reversal training; CNO was administered 30 min before each reversal training session. Note that in this instrumental task, the action-outcome retrieval sequence was also switched at reversal. During initial training, rewards were presented adjacent to the nose-poke port. Whereas during reversal training, rewards were presented on the wall opposite to where the nose-poke port was. During initial training (A1→O1), nose-poke rates and magazine entries did not differ between groups (Supplementary Fig. 6e; $F_{(1,17)} = 0.694$, $p = 0.416$, Supplementary Fig. 7b; $F_{(1,17)} = 0.186$, $p = 0.672$), and both groups were sensitive to food devaluation (Supplementary Fig. 6f, mCh: $t_{(7)} = 6.169$, hM4Di: $t_{(9)} = 5.519$). On the first day of reversal training (A2→O1), although nose-pokes and rewards earned did not differ between groups, the hM4Di group had more magazine entries than controls (nose-pokes: Supplementary Fig. 6g, $t_{(16)}$ = -

1.076, p = 0.298; rewards: Supplementary Fig. 6h, $t_{(15)}$ = -0.781, p = 0.447; entries: Supplementary Fig. 6i, $t_{(16)}$ = -2.334; nose-pokes/min: Supplementary Fig. 7c, $F_{(1,17)}$ = 0.0007, p = 0.979; entries (O1): Supplementary Fig. 7d, $t_{(17)}$ = 2.114; entries (O2): Supplementary Fig. 7e, $F_{(1,17)}$ = 0.006, p = 0.937). The return map of inter-entry intervals was found to be more scattered for controls but was concentrated near the origin for the hM4Di group (Supplementary Fig. 6j, k), suggesting that the hM4Di group entered the magazine more frequently. Each data point signifies the time delay to its preceding (x) and succeeding (y) magazine entry. Consequently, the mean inter-entry interval was shorter in the hM4Di group than in controls (Supplementary Fig. 6l, $t_{(18)}$ = 2.125), suggesting that chemogenetic CIN inhibition caused frequent excessive magazine entries to check for reward upon contingency reversal [1, 2].

To investigate any differences in the performance of the reversed action-outcome association between groups, we identified those magazine entries (O1) that were preceded (within 10 sec) by a nose-poke (A2, Supplementary Fig. 6m, n). We found that such entries were more condensed within the first 200 entries in the control group than in the hM4Di group (Supplementary Fig. 6o, $t_{(14)}$ = 2.24). Given that both groups eventually learned the reversed action-outcome associations (Supplementary Fig. 7f; Ctrl: $t_{(6)}$ = 2.391, hM4DI: $t_{(8)}$ = 4.579), this result suggests that chemogenetic CIN inhibition slowed down the reversal learning process.

Next, we analyzed the reward-taking behavior for the two groups during reversal training. In a typical bout, an animal first nose-pokes to trigger reward delivery, then enters the magazine to collect it (Supplementary Fig. 6p). We discovered that the control animals, but not the hM4Di group, entered the magazine to collect rewards immediately

after delivery during reversal training (Supplementary Fig. 6q, r; $F_{(1,20)} = 6.784$). We calculated the percentage of rewards collected (reward delivery followed by at least 1 magazine entry) over total rewards delivered and found that although there was no difference between groups on the last day of initial training (Supplementary Fig. 6r, Ini; $p = 0.346$), the percentages on the first three reversal training sessions were lower in the hM4Di group than mCherry controls. This result indicates that chemogenetic CIN inhibition impairs reward collection during reversal training. [2]

We found that chemogenetic inhibition of DMS CINs transiently led to 1) frequent, excessive magazine entries, 2) slowed reversal learning, and 3) impaired reward collection upon contingency reversal. Additionally, time-locked optogenetic CIN inhibition during reversal training also led to frequent excessive magazine entries, and impaired reversal and extinction learning. Interestingly, animals eventually learned the reversed action-outcome associations after chemogenetic, but not optogenetic, CIN inhibition. This discrepancy may result from 1) Repeated CNO injections may cause adaptations and receptor desensitization that could compromise CNO-induced CIN inhibition during reversal days 2-4 [3, 4]. 2) The spatial shift of the action-outcome retrieval sequence is more salient in the chemogenetic (A-O on opposite walls) versus optogenetic (A-O on the same wall) experiment. Salient changes induce strong thalamic inputs to cause CIN bursting [5, 6], which could subdue hM4Di (Gi)-mediated chemogenetic inhibition. Taken together, our study provides evidence that DMS CIN activity during reward delivery mediates behavioral flexibility.

Supplementary References

1. Son, J.H., Kuhn, J. & Keefe, K.A. Perseverative behavior in rats with methamphetamine-induced neurotoxicity. *Neuropharmacology* **67**, 95-103 (2013).
2. Lhost, J.*, et al.* Interplay Between Inhibitory Control and Behavioural Flexibility: Impact of Dorsomedial Striatal Dopamine Denervation in Mice. *Neuroscience* **477**, 25-39 (2021).
3. Gomez, J.L.*, et al.* Chemogenetics revealed: DREADD occupancy and activation via converted clozapine. *Science* **357**, 503-507 (2017).
4. Claes, M., De Groef, L. & Moons, L. The DREADDful Hurdles and Opportunities of the Chronic Chemogenetic Toolbox. *Cells* **11**, 1110 (2022).
5. Bradfield, L.A., Bertran-Gonzalez, J., Chieng, B. & Balleine, B.W. The thalamostriatal pathway and cholinergic control of goal-directed action: interlacing new with existing learning in the striatum. *Neuron* **79**, 153-166 (2013).
6. Ding, J.B., Guzman, J.N., Peterson, J.D., Goldberg, J.A. & Surmeier, D.J. Thalamic gating of corticostriatal signaling by cholinergic interneurons. *Neuron* **67**, 294-307 (2010).
