# Supplementary material for: Drug reinforcement impairs cognitive flexibility by inhibiting striatal cholinergic neurons
Source: Nat Commun. 2023 Jun 30;14:3886. doi: 10.1038/s41467-023-39623-x (PMC10313783; doi:10.1038/s41467-023-39623-x)
Supplement: Supplementary file 3 — Reporting Summary [file 41467_2023_39623_MOESM3_ESM.pdf]

nature portfolio

Corresponding author(s): Jun Wang

Last updated by author(s): May, 26, 2023

Reporting Summary

Nature Portfolio wishes to improve the reproducibility of the work that we publish. This form provides structure for consistency and transparency in reporting. For further information on Nature Portfolio policies, see our Editorial Policies and the Editorial Policy Checklist.

Statistics

For all statistical analyses, confirm that the following items are present in the figure legend, table legend, main text, or Methods section.

n/a	Confirmed	
☐	☒	The exact sample size (n) for each experimental group/condition, given as a discrete number and unit of measurement
☐	☒	A statement on whether measurements were taken from distinct samples or whether the same sample was measured repeatedly
☐	☒	The statistical test(s) used AND whether they are one- or two-sided<br>*Only common tests should be described solely by name; describe more complex techniques in the Methods section.*
☐	☒	A description of all covariates tested
☐	☒	A description of any assumptions or corrections, such as tests of normality and adjustment for multiple comparisons
☐	☒	A full description of the statistical parameters including central tendency (e.g. means) or other basic estimates (e.g. regression coefficient) AND variation (e.g. standard deviation) or associated estimates of uncertainty (e.g. confidence intervals)
☐	☒	For null hypothesis testing, the test statistic (e.g. F, t, r) with confidence intervals, effect sizes, degrees of freedom and P value noted<br>*Give P values as exact values whenever suitable.*
☒	☐	For Bayesian analysis, information on the choice of priors and Markov chain Monte Carlo settings
☒	☐	For hierarchical and complex designs, identification of the appropriate level for tests and full reporting of outcomes
☒	☐	Estimates of effect sizes (e.g. Cohen's d, Pearson's r), indicating how they were calculated

Our web collection on statistics for biologists contains articles on many of the points above.

Software and code

Policy information about availability of computer code

Data collection	All software used for data collection is commercially available. For rat and mice operant testing, MED-PC V (Med Associates), Graphic State 4.0 (Coulbourn Instruments) was used. Electrophysiology data were acquired using Clampex (in pClamp 10.7, Molecular Devices). For slice imaging, data were acquired with FluoView1200 (Olympus). Fiber-photometry data was collected using OceanView 1.6.7.
Data analysis	Electrophysiology data was analyzed using using Clampfit (in pClamp 10.7, Molecular Devices) and Mini Analysis (Mini60, Synaptosoft Inc.). Slice images were analyzed using Imaris 8.3.1 (Bitplane, Zurich, Switzerland). Data were graphed using Origin Pro 2019 (Origin lab) and statistics were conducted using SigmaPlot 12.0 (Systat Software Inc.) and SPSS 29.0. Finalized data was organized in MS Excel 16.0. The code used for analysis of fiber photometry data in the current study is available online at the Zenodo public repository [https://doi.org/10.5281/zenodo.7948766] and is also referenced in the manuscript.

For manuscripts utilizing custom algorithms or software that are central to the research but not yet described in published literature, software must be made available to editors and reviewers. We strongly encourage code deposition in a community repository (e.g. GitHub). See the Nature Portfolio guidelines for submitting code & software for further information.

Data

Policy information about availability of data

All manuscripts must include a data availability statement. This statement should provide the following information, where applicable:

- Accession codes, unique identifiers, or web links for publicly available datasets
- A description of any restrictions on data availability
- For clinical datasets or third party data, please ensure that the statement adheres to our policy

Source data are provided with this paper. Coordinates for stereotaxic surgeries were derived from the mouse and rat brain atlas (Franklin et al, 2007;Paxinos et al 2007).

Human research participants

Policy information about studies involving human research participants and Sex and Gender in Research.

Reporting on sex and gender	not applicable
Population characteristics	not applicable
Recruitment	not applicable
Ethics oversight	not applicable

Note that full information on the approval of the study protocol must also be provided in the manuscript.

Field-specific reporting

Please select the one below that is the best fit for your research. If you are not sure, read the appropriate sections before making your selection.

[x] Life sciences [ ] Behavioural & social sciences [ ] Ecological, evolutionary & environmental sciences

For a reference copy of the document with all sections, see nature.com/documents/nr-reporting-summary-flat.pdf

Life sciences study design

All studies must disclose on these points even when the disclosure is negative.

Sample size	Sample sizes were estimated using SigmaPlot 12.0 software. Expected difference in means, expected standard deviation and desired alpha value were fed into the statistical test sample size calculator, which returned the estimated sample size.
Data exclusions	We indicate that animals with virus mis-injections or head cap loss, or lack of responding in the operant setting were excluded from analysis. Outliers and unstable electrophyisiology recordings with a change in series resistance of more than 10% were excluded from the analyses.
Replication	All experiments were done in triplicate and reliably reproduced.
Randomization	All animals were randomly allocated into experimental and control groups and counterbalanced across groups.
Blinding	Experimenter was blinded during data collection and data analysis for all experiments. Animal subjects were given IDs independent of treatment groups and data was organized into their respective groups only after the analysis was finished.

Reporting for specific materials, systems and methods

We require information from authors about some types of materials, experimental systems and methods used in many studies. Here, indicate whether each material, system or method listed is relevant to your study. If you are not sure if a list item applies to your research, read the appropriate section before selecting a response.

Materials & experimental systems

n/a	Involved in the study
☐	☒ Antibodies
☒	☐ Eukaryotic cell lines
☒	☐ Palaeontology and archaeology
☐	☒ Animals and other organisms
☒	☐ Clinical data
☒	☐ Dual use research of concern

Methods

n/a	Involved in the study
☒	☐ ChIP-seq
☒	☐ Flow cytometry
☒	☐ MRI-based neuroimaging

Antibodies

Antibodies used

anti-ChAT antibody (EMD milii pore AB144P, LOT #2947408, Dil. 1:200 (2 nights)), donkey anti-goat antibody (Invitrogen A21447, LOT #2273668, Dil. 1: 500 (2 nights)).

Validation

AB144P: Saunders et al 2015, PMID: 25723967. A21447: Jae et al 2016, PMID: 27793667.

Animals and other research organisms

Policy information about studies involving animals; ARRIVE guidelines recommended for reporting animal research, and Sex and Gender in Research

Laboratory animals

Mice: C57BL/6J, ChAT-Cre(+/+), deltaneoChAT-Cre(+/+), ChATeGFP(+/+), Ai14(+/+), DltdTomato(+/-), Ai32(+/+), Drdl-Cre(+/-), Ai167 (+/-), A2A-ere(+/-) Rats: D1-Cre(+/-), ChAT-Cre(+/-), tdTomato/Hom-KI(+/+) All experiments were conducted in 4- to 7-month-old animals. Both mice (4-5 mice per cage) and rats (2 rats per cage) were group-housed and maintained in a temperature (72 degree Fahrenheit)- and humidity (54%)-controlled environment.

Wild animals

Study did not include wild animals.

Reporting on sex

All electrophysiology, histology and behavioral experiments were conducted in both male and female mice/rats. In total, 116 male and 82 female animals were used for the study, sex was not considered in the study design. This was because Jentsch et al, 2002 (Neuropsychopharmacology) found that cocaine administration impaired reversal learning in both male and female animals equally.

Field-collected samples

Study did not include samples collected from the field.

Ethics oversight

All animal care and experimental procedures were approved by Texas A&M University Animal Care and Use Committee and were conducted in agreement with the National Research Council Guide for the Care and Use of Laboratory Animals All animal care and experimental procedures were approved by the Texas A&M University Institutional Animal Care and Use Committee and were conducted in agreement with the National Research Council Guide for the Care and Use of Laboratory Animals (AUP# 2022-0198).

Note that full information on the approval of the study protocol must also be provided in the manuscript.
